# Expression of the epidermal growth factor receptor (EGFR) and the phosphorylated EGFR in invasive breast carcinomas

**DOI:** 10.1186/bcr2103

**Published:** 2008-06-03

**Authors:** Christina Magkou, Lydia Nakopoulou, Christina Zoubouli, Kanelina Karali, Irene Theohari, Panagiotis Bakarakos, Ioanna Giannopoulou

**Affiliations:** 1 First Department of Pathology, School of Medicine, National and Kapodistrian University of Athens, Mikras Asias, Athens, 11527, Greece; 2 Biology Department, School of Sciences, National and Kapodistrian University of Athens, Panepistimioupolis Zografou, Athens, 15784, Greece

## Abstract

**Introduction:**

Epidermal growth factor receptor (EGFR) is involved in regulating cell growth in breast carcinomas. Its activated form, phosphorylated EGFR (pEGFR), is correlated with poor prognosis in lung cancer, but it has not yet been fully investigated in breast cancer. The aim of this study was to investigate the expressions of EGFR and pEGFR and their correlation with overall and disease-free survival, clinicopathological parameters and biological markers of invasion and angiogenesis (phosphorylated Akt [pAkt], urokinase plasminogen activator receptor [uPAR], matrix metalloproteinase [MMP]-14, vascular endothelial growth factor receptor [VEGFR]-1/Flt-1).

**Methods:**

A three-step immunohistochemical method was applied to paraffin-embedded sections from 154 patients with invasive breast carcinoma in order to detect expressions of the proteins EGFR, pEGFR, oestrogen receptor, progesterone receptor, c-erbB-2, pAkt, VEGFR-1/Flt-1, MMP-14 and uPAR. The results were evaluated statistically using the χ^2 ^test. Overall and disease-free survival distribution curves were assessed using the Kaplan-Meier test and log-rank statistics, followed by Cox proportional hazards regression model.

**Results:**

EGFR and pEGFR proteins were immunodetected in the membrane of the malignant cells (11.3% and 35.7%, respectively). EGFR expression was positively correlated with nuclear grade (*P *= 0.001) and negatively correlated with the hormonal receptor oestrogen receptor (*P *= 0.005). pEGFR was positively related to the Akt pathway (*P *= 0.008) and appeared to participate in invasion and metastasis (uPAR, *P *= 0.049; MMP-14, *P *= 0.025; VEGFR-1/Flt-1, *P *= 0.016). Univariate analysis showed that the EGFR/pEGFR phenotype was associated with poor overall survival (*P *= 0.019), a finding further supported by multivariate analysis (*P *= 0.013).

**Conclusion:**

These data provide evidence that pEGFR expression is related to angiogenesis (via VEGFR-1/Flt-1, MMP-14 and pAkt pathways) and invasiveness (via uPAR, MMP-14 and pAkt pathways) and that the EGFR/pEGFR phenotype is associated with poor patient survival in invasive breast cancer.

## Introduction

Epidermal growth factor receptor (EGFR) is a member of the ErbB family, a family of tyrosine kinase receptors with growth-promoting effects [1]. It is expressed in several carcinomas [2,3] and high levels of expression are a common feature of the malignant phenotype in many solid human tumours [4]. It is activated by up to four different ligands, most commonly epidermal growth factor (EGF) and transforming growth factor-α(TGF-α), and forms homodimeric complexes or heterodimeric complexes with other members of the ErbB family of receptors. Ligand binding and dimerization causes autophosphorylation of the intracytoplasmic domains and activation of the intracellular tyrosine kinase. Activated EGFR (phosphorylated EGFR [pEGFR]) stimulates a number of different signal transduction pathways, such as the Ras/mitogen-activated protein kinase pathway, the phosphoinositide-3 kinase (PI3K)/Akt pathway and the phospholipase-Cγ/protein kinase C pathway. The activation of these pathways initialize with the recruitment of different adaptor proteins, which bind to different phosphotyrosine residues of the cytopasmic tail of EGFR, and continues with a highly complex network of enzymes, proteins and small-molecule secondary messengers [5]. The signal transduction pathways activated by pEGFR play important roles in various cellular processes, such as cell proliferation, differentiation, adhesion, migration and apoptosis [4,6].

EGFR is reported to be expressed in 14% to 91% of patients with breast cancer [7-12], and in several studies it has also been associated with poor prognosis [7], although its prognostic value remains unclear. Recently, EGFR has once again come to the fore, because of the development of several novel drugs that target EGFR. Because EGFR has not proven to be a useful prognostic/predictive marker of clinical response to EGFR-targeted therapies [9,13], other prognostic/predictive markers have been proposed, including the activated form of EGFR (pEGFR) [14]. Previously, EGFR phosphorylation was found to be associated with poor prognosis in non-small-cell lung cancer patients and was suggested to be an important predictor of clinical outcome [15,16].

The purpose of the present study was to gain further insight into the prognostic significance of EGFR and pEGFR expression in invasive breast cancer. This was assessed in relation to the classical clinicopathological parameters, clinical outcome and the expression of biological markers of invasion and angiogenesis (phosphorylated Akt [pAkt], urokinase plasminogen activator receptor [uPAR], matrix metalloproteinase [MMP]-14 and vascular endothelial growth factor receptor [VEGFR]-1/Flt-1).

## Materials and methods

### Patients and samples studied

A total of 154 paraffin blocks incorporating tumour samples were available from patients with resectable breast cancer and who had undergone surgery. We only selected women with histologically proven, clearly invasive breast carcinomas, regardless of their initial stage, in whom axillary lymph node dissection had been performed and whose resected material was studied histologically. The patients were aged 25 to 86 years (mean age 57.12 years). None of them had received radiation or chemotherapy preoperatively. Finally, the material acquired from them was used in research after they had provided informed consent, and we also obtained the institution's approval before performing the study.

Routine histological examination was performed with haematoxylin-eosin staining. All carcinomas were classified according to the criteria of the World Health Organization [17] and were recorded as invasive ductal or invasive lobular. The combined histological grade (1, 2 and 3) of infiltrating ductal carcinoma was obtained in accordance with a modified Scarff-Bloom-Richardson histologic grading system, with guidelines as suggested by Nottingham City Hospital pathologists [18]. Nuclear grading was based on nuclear polymorphism and mitotic activity. Staging at the time of diagnosis was based on the TNM (tumour-node-metastasis) system [19]. Tumour size (<2 cm, 2 to 5 cm, >5 cm) and lymph node status were evaluated separately. The clinicopathological characteristics of the series are shown in Table 1.

**Table 1:** Correlation of EGFR and pEGFR expression with clinicopathological parameters, ER/PR, c-erbB-2, pAkt, uPAR, MMP-14 and VEGFR-1/Flt-1

Parameters		Total	EGFR		PEGFR	
			
			*n *(%)	*P*	*n *(%)	*P*
Menopausal status	Premenopausal	50	5 (10.0)	NS	13 (26.0)	NS
	Postmenopausal	104	13 (12.5)		42 (40.4)	
Histological type	Ductal	121	15 (12.4)	NS	41 (33.9)	NS
	Lobular	32	3 (9.4)		13 (40.6)	
Histological grade	1	23	1 (4.3)	NS	7 (30.4)	NS
	2	90	11 (12.2)		35 (38.9)	
	3	36	6 (16.7)		11 (30.6)	
Nuclear grade	1	53	1 (1.9)	0.001	18 (34.0)	NS
	2	53	5 (9.4)		23 (43.4)	
	3	47	12 (25.5)		13 (27.7)	
Tumour size	<2 cm	37	2 (5.4)	NS	13 (35.1)	NS
	2 to 5 cm	91	11 (12.1)		32 (35.2)	
	>5 cm	25	5 (20.0)		9 (36.0)	
Lymph node status	Noninfiltrated	61	6 (9.8)	NS	19 (31.1)	NS
	Infiltrated	91	12 (13.2)		35 (38.5)	
Stage	1	28	2 (7.1)	NS	9 (32.1)	NS
	2	97	12 (12.4)		33 (34.0)	
	3	27	4 (14.8)		12 (44.4)	
ER	Negative	70	14 (20.0)	0.005	20 (28.6)	NS
	Positive	83	4 (4.8)		35 (42.2)	
PR	Negative	78	13 (16.7)	NS	30 (38.5)	NS
	Positive	75	5 (6.7)		25 (33.3)	
c-erbB-2	Negative(<10)	60	5 (8.3)	NS	21 (35.0)	NS
	Positive(≥ 10)	93	13 (14.0)		34 (36.6)	
pAkt	Negative (<10)	49	3 (6.1)	NS	12 (24.5)	0.008
	Positive (≥ 10)	72	11 (15.3)		35 (48.6)	
uPAR	Negative (<15)	49	9 (18.3)	NS	12 (24.5)	0.049
	Positive (≥ 15)	67	6 (9.0)		29 (43.3)	
MMP-14	Negative (= 0)	93	13 (14.0)	NS	28 (30.1)	0.025
	Positive (>0)	29	2 (6.9)		16 (55.2)	
VEFGR-1/Flt-1	Negative (<30)	40	5 (12.5)	NS	9 (22.5)	0.016
	Positive (≥ 30)	72	9 (12.5)		33 (45.8)	

EGFR, epidermal growth factor receptor; ER, oestrogen receptor; MMP, matrix metalloproteinase; NS, not significant; pAkt, phosphorylated Akt; pEGFR, phosphorylated epidermal growth factor receptor; PR, progesterone receptor; uPAR, urokinase plasminogen activator receptor; VEGFR, vascular endothelial growth factor receptor.

Follow up was available for 151 patients, of whom 39 died from breast cancer and 54 had a recurrence. Mean survival time was 94.83 months (range 5 to 135 months). Patient outcome was defined as disease-free and overall survival rates. Depending on the extent of their disease, all patients received conventional postoperative treatment, including radiation therapy and medical anti-oestrogen therapy, when indicated. Premenopausal patients with axillary involvement were treated with six courses of adjuvant chemotherapy.

### Immunohistochemistry

Immunohistochemical staining for EGFR and pEGFR was performed on 4 μm thick formalin-fixed paraffin sections, after overnight heating at 37°C. Subsequent deparaffinization, rehydration and antigen retrieval were performed in a one-step procedure with the EDTA (pH 8.0; Trilogy, Cell Marque, Rocklin, CA, USA) in a microwave oven by heating the slides for 15 minutes. After rinsing with Tris-buffered saline, normal horse serum was applied for 30 minutes to block nonspecific antibody binding. Subsequently, sections were incubated overnight at 4°C with the primary antibody. A three-step technique (Elite ABC Vector Laboratories, Burlingame, CA, USA) was used for visualization, with diaminobenzidine as a chromogen. Finally, sections were counterstained with haematoxylin and mounted.

A mouse antihuman monoclonal antibody against EGFR (clone 2-18C9, #1492; Dako, Glostrup, Denmark), ready for use, and a rabbit monoclonal antibody against the pEGFR (Tyr 1173, #4407; Cell Signaling Technology, Beverly, MA, USA) was used at a dilution of 1:100. According to the manufacturer, the anti-pEGFR antibody applied in the study does not crossreact with other phosphorylated receptors of the HER (human epidermal growth factor receptor) family. It detects endogenous levels of EGFR only when it is phosphorylated at tyrosine 1173; therefore, it is clear that this antibody targets the pEGFR epitope.

The immunomarkers assessed in the present study, in combination with EGFR and pEGFR, had previously been detected using the following antibodies: anti-oestrogen receptor (ER) clone 1D5 (Dako) at a dilution of 1:450; anti-progesterone receptor (PR) clone 1A6 (Dako) at a dilution of 1:150; anti-c-erbB-2 clone BP53.12 (Oncogene, Cambridge, MA, USA) at a dilution of 1:150; anti-pAkt (Thr308) rabbit monoclonal antibody (244F9, #4056; Cell Signaling Technology) at a dilution of 1:40; anti-uPAR mouse antihuman domain 2 monoclonal antibody (American Diagnostic Inc., Greenwich, CT, USA) at a dilution of 1:100; anti-MMP-14 polyclonal antibody (Neomarkers, Fremont, CA, USA) at a dilution of 1:120; and anti-Flt-1 (VEGFR-1) rabbit polyclonal antibody (C-17) sc316 (Santa Cruz Biotechnology Inc., CA, USA) at a dilution of 1:80. The results with the aforementioned immunomarkers were obtained from our archival database.

### Evaluation of immunohistochemistry

The evaluation of the immunohistochemical staining was performed by two pathologists, independently, through light microscopic observation and without knowledge of the clinical data from each patient. Cases of disagreement were reviewed jointly to reach a consensus score. Evaluation was performed by using the scoring system of Putti and coworkers [20] with slight modifications. The extent of EGFR or pEGFR immunoreactivity was scored as 0 points for less than 5% positive cells, 1 point for 5% to 9% positive cells, 2 points for 10% to 50% positive cells, and 3 points for more than 50% positive cells. The intensity of EGFR or pEGFR immunoreactivity was scored as 1 point for weak staining, 2 points for moderate staining, and 3 points for strong staining. The overall score of the staining for each case was obtained by multiplying the extent of immunoreactivity score with the intensity score. Cases with overall score greater than or equal to 1 were considered positive. With regard to positive control, we used a breast cancer tissue section previously known to over-express EGFR and pEGFR (external control), along with a sample adjacent to cancerous tissues (internal staining control). Negative controls had the primary antibody omitted and replaced by nonimmune normal serum from the same species as the primary antibody or Tris-buffered saline.

Staining for ER and PR was evaluated semiquantitatively using the H score system, and a score greater than 50 was considered to be positive for both antigens [18]. The evaluation of the immunostaining of anti-ER, anti-PR, anti-cerbB-2, anti-pAkt, anti-uPAR, anti-MMP14 and anti-Flt-1 was performed, as described previously [21-23]. The cut-off values of these immunomarkers are shown in Table 1.

### Statistical analysis

The significance of the relationship between the expression of EGFR and pEGFR and clinicopathological parameters was evaluated with univariate analysis using χ^2 ^test and Fisher's exact probability test. The effect of EGFR and pEGFR differential expression on postoperative survival rates was assessed using both univariate (log-rank test) and multivariate (stepwise forward Cox's proportional hazard regression model) analysis. A *P *value under 0.05 was regarded to indicate statistical significance.

## Results

**Figure 1:**
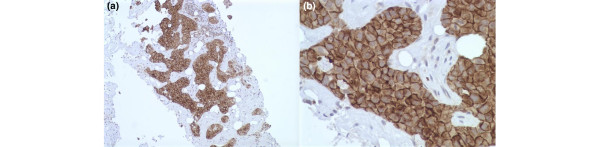
Immunohistochemisty of EGFR and pEGFR in invasive breast carcinoma. Intense membrane staining of **(a) **EGFR (ABC/HRPX200) and of **(b) **pEGFR (ABC/HRPX400) in two cases of invasive ductal breast carcinoma. EGFR, epidermal growth factor receptor; pEGFR, phosphorylated epidermal growth factor receptor.

EGFR and pEGFR proteins were immunodetected in the membrane of the malignant cells in 18 (11.3%) and 55 cases (35.7%), respectively (Figure 1). Of 154 patients, eight (5.2%) were both EGFR and pEGFR positive, 10 (6.5%) were EGFR positive only, 47 (30.5%) were pEGFR positive only, and 89 (57.8%) were completely negative.

Based on expressions of EGFR, ER and c-erbB-2, only two carcinomas were classified as breast carcinomas of basal-like phenotype (EGFR-positive, ER-negative and c-erbB-2 negative). None of the studied proteins was associated with menopausal status, tumour size, lymph node status, tumour stage, PR hormonal status and c-erbB-2 protein.

EGFR immunoexpression was related to nuclear grade (*P *= 0.001) and inversely related to ER receptor status (*P *= 0.005). Immunoexpression of pEGFR was associated with pAkt, uPAR, MMP-14 and VEGFR-1/Flt-1 (*P *= 0.008, *P *= 0.049, *P *= 0.025 and *P *= 0.016, respectively). Coexpression of EGFR/pEGFR was statistically associated with pAkt, MMP-14 and VEGFR-1/Flt-1 (*P *= 0.011, *P *= 0.039 and *P *= 0.019, respectively).

**Figure 2:**
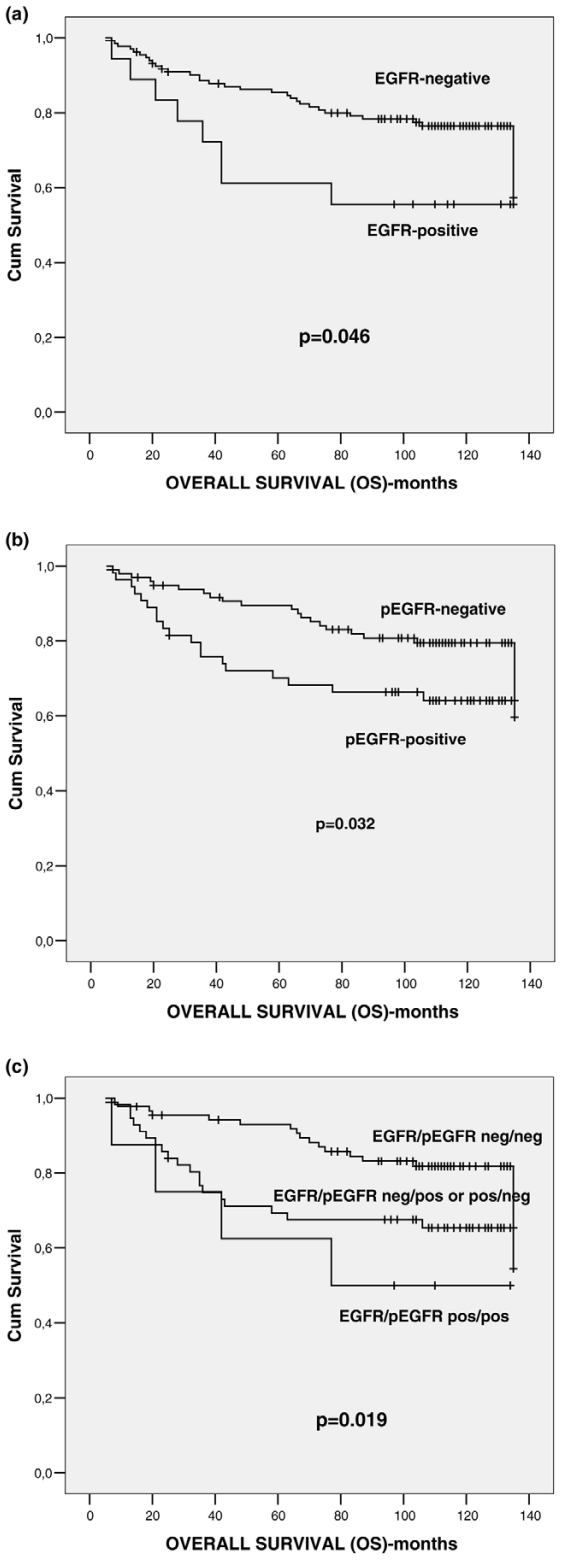
Univariate analysis (log-rank test) of overall survival. Schematic representation of the impact of **(a) **EGFR, **(b) **pEGFR and **(c) **EGFR/pEGFR expression on overall survival (OS). EGFR, epidermal growth factor receptor; pEGFR, phosphorylated epidermal growth factor receptor.

With regard to their prognostic significance, EGFR and pEGFR were found to have an unfavourable associated with overall survival (*P *= 0.046 and *P *= 0.032, respectively), but not with disease-free survival (univariate analysis). Interestingly, patients with synchronous expression of EGFR and pEGFR had worse prognosis (*P *= 0.019) than did EGFR-positive or pEGFR-positive patients and totally negative patients (Figure 2). Moreover, in multivariate analysis adjusted for numerous factors (age, menopausal status, histological type, histological grade, tumour size, lymph node status, ER, PR, c-erbB-2, EGFR, pEGFR and EGFR/pEGFR phenotype), tumour size (*P *= 0.010; B coefficient = 6.550; standard error = 0.728, 95% confidence interval = 1.573 to 27.281) and EGFR/pEGFR coexpression (*P *= 0.013; B coefficient = 1.520; standard error = 0.168, 95% confidence interval = 1.093 to 2.113) were of independent prognostic significance for overall survival.

## Discussion

This study examined the expression of EGFR and its activated form, pEGFR, in invasive breast carcinoma. We demonstrated that EGFR and pEGFR are both expressed in the membrane of 11.3% and 35.7% of tumour cells. The greater rate of positivity for pEGFR than for EGFR could be attributed to the EGFR downregulation process. Once EGFR is activated it undergoes internalization, resulting in a marked decrease in nonactivated membrane-bound EGFR [24]. In previous studies, EGFR immunopositivity exhibits great variety [7-12]. The wide range of EGFR expression (14% to 91%) may be accounted for by use of different methods and different criteria for assessment, as well by the presence of basal-like carcinomas that consistently over-express EGFR.

None of the studied proteins was associated with menopausal status, tumor size, lymph node status, tumour stage and PR hormonal status, which is in accordance with the majority of reports on EGFR expression [9,25]. The absence of any association between EGFR and c-erbB-2 expression may be due to variations in c-erbB-4 expression, which antagonizes the influence of c-erbB-2 in tumors [7].

With regard to EGFR, we showed that its expression was positively associated with nuclear grade (*P *= 0.001) and inversely associated with ER hormonal status (*P *= 0.005), which is in accordance with a large number of studies [25,26]. This consistent finding in much of the literature had led to the plausible hypothesis that EGFR signalling may be associated with endocrine resistance or insensitivity [27-29]. Alternatively, this finding may be accounted for by the strong association between EGFR expression and loss of differentiation in breast cancer.

EGFR activation has an antiapoptotic effect through PI3K pathway [1], a fact that was corroborated by the pEGFR relationship to pAkt (*P *= 0.014). EGFR can lead to activation of PI3K both directly and indirectly through Ras; it induces downstream activation of phosphoinositide-dependent protein kinase-1 and -2 that phosphorylate thr308 and ser473 on Akt, respectively. Akt's antiapoptotic role is well known, via phosphorylating and sequestering downstream targets including the FOXO family of forkhead transcription factors, the proapoptotic Bad and the protease caspase-9, and by activating the pro-survival transcriptional regulator protein nuclear factor-κB.

Expression of pEGFR was associated with VEGFR-1/Flt-1 and MMP-14. VEGFR-1/Flt-1 is a specific endothelial cell receptor to which the angiogenic factors VEGF-A and VEGF-B bind; it promotes differentiation and vascular maintenance [30]. Indeed, in tumour progression EGFR upregulates VEGFR; thus, it is implicated in angiogenesis. In addition, in human cancer cells the EGFR autocrine pathway controls the production of several proangiogenic growth factors, including VEGF [4]. It is also known that several EGFR inhibitors, such as monoclonal antibodies, result in a concurrent downregulation of tumour-induced, VEGF-mediated angiogenesis [4]. Therefore, the above-mentioned relationship implies an angiogenetic role of activated EGFR. The angiogenetic ability of pEGFR has further confirmed by the pEGFR association with pAkt, which is known to modulate angiogenesis via activation of endothelial nitric oxide synthase [31]. MMP-14, as well as most of the MMPs, may promote angiogenesis by at least two different mechanisms: by degrading barriers and allowing endothelial cell invasion; or by liberating factors that promote or maintain the angiogenic phenotype [32]. In addition, it promotes invasion and metastasis by degrading ectracellular matrix [33]. It appears that the above-mentioned relationship between EGFR and MMP-14 reflects the well established interaction between EGFR pathway and the MMPs [1]. EGFR activation is able to upregulate MMPs, whereas MMPs participate in several pathways of EGFR activation, such as the ectodomain shedding of EGFR transmembrane precursor [4], G protein-coupled receptor-mediated transactivation, and uPAR-mediated transactivation of EGFR [1].

The combination of EGFR/pEGFR was associated with pAkt, MMP-14 and VEGFR-1, which may be accounted for by the parallel relationship between pEGFR and those biological parameters.

Another observation in the present study, that of the parallel relationship between pEGFR and uPAR, supports the existence of a uPAR-mediated EGFR transactivation pathway [1] and enforces the invasive effect of pEGFR. The uPAR glycoprotein, receptor of plasminogen activation system, plays a central role in extracellular matrix degradation; thus, it participates in tumour invasion and metastasis [34]. Previously, in breast cancer, it has been related to tumour aggressiveness and patients poor survival [21].

With regard to the association of EGFR and pEGFR with prognosis and survival, we found that EGFR and its active form are significantly associated with overall survival (*P *= 0.035 and *P *= 0.016, respectively) but not with disease-free survival in univariate analysis. Findings from previous studies regarding prognostic significance of EGFR are controversial. Tsuitsu and coworkers [11] reported the prognostic value of EGFR for overall and disease-free survival, whereas other investigators failed to confirm its prognostic significance in either the entirety of studied cases [7-9] or in lymph node positive [8] or -negative cases [35,36]. On the other hand, there is no report about pEGFR expression and prognosis in breast cancer, as far as we know, whereas in our study pEGFR appeared to have an unfavourable impact on overall survival. We also observed that EGFR-positive/pEGFR-positive tumours had worse prognosis in contrast to EGFR-negative and/or pEGFR-negative ones. Moreover, EGFR/pEGFR was found to be an independent prognostic indicator for overall survival. EGFR and pEGFR coexpression appears to be more representative of EGFR dynamics in tumour than solely EGFR or pEGFR expression. Probably, in EGFR-positive/pEGFR-positive tumours, the total number of receptors is greater; thus, there are more receptors available for subsequent phosphorylation. Another possible explanation might be the existence of an inhibitory mechanism, which impedes autophosphorylation of several receptors and permits only a few receptors to become phosphorylated and initiate EGFR-dependent signalling cascades.

Nieto and coworkers [37] also studied EGFR and pEGFR expression in invasive breast cancer, but they failed to attribute any prognostic value to pEGFR expression. To the best of our knowledge, there are no published data confirming the prognostic value of pEGFR or EGFR/pEGFR coexpression in breast cancer. Interestingly, Arteaga and Baselga [14] have noticed that EGFR-positive colon carcinomas with simultaneous expression of pEGFR, coexpressed the EGFR ligand transforming growth factor-α and markers of tumour proliferation, which is in contrast to EGFR-positive/pEGFR-negative carcinomas. Thus, EGFR content in EGFR-positive/pEGFR-negative tumours does not reflect the level of receptor activation.

## Conclusion

In the present study, simultaneous expression of both forms of EGFR emerged as a more promising prognostic marker in invasive breast carcinomas. However, more research is needed to clarify the importance of EGFR/pEGFR immunoexpression to prognosis, as well as the applicability of pEGFR expression and amplification in targeted therapies.

## Abbreviations

EGFR = epidermal growth factor receptor; ER = oestrogen receptor; MMP = matrix metalloproteinase; pAkt = phosphorylated Akt; pEGFR = phosphorylated epidermal growth factor receptor; PI3K = phosphoinositide-3 kinase; PR = progesterone receptor; uPAR = urokinase plasminogen activator receptor; VEGFR = vascular endothelial growth factor receptor.

## Competing interests

The authors declare that they have no competing interests.

## Authors' contributions

CM was responsible for the statistical analysis and the manuscript preparation. LN provided overall guidance, direction and critical review of the study design. KK was responsible for performing the immunohistochemical protocols, manuscript preparation, data collection and editing. CZ and IT were responsible for the evaluation of the immunohistochemical staining. PB was responsible for data collection. IG was responsible for supervising the immunohistochemical protocols and editing.
